# Resistance training increases fibroblast growth factor-21 and irisin levels in the skeletal muscle of Zucker diabetic fatty rats

**DOI:** 10.20463/jenb.2017.0008

**Published:** 2017-09-30

**Authors:** Hee-jae Kim, Wook Song

**Affiliations:** 1. Physical activity and performance institute, Konkuk University, Seoul Republic of Korea; 2. Health and Exercise Science, Institute of sport science, Seoul National University, Seoul Republic of Korea; 3. Institute on Aging, Seoul National University, Seoul Republic of Korea

**Keywords:** FGF-21, Irisin, Resistance training, Diabetes, Zucker diabetic fatty rat

## Abstract

**[Purpose]:**

Although the fibroblast growth factor-21 (FGF-21) and irisin roles are well demonstrated in metabolic disease, there have been no reports investigating the effect of resistance exercise on FGF-21 and irisin levels in diabetic skeletal muscles. Therefore, this study aimed to investigate the change of FGF-21 and irisin levels in various skeletal muscles, and their association with muscle strength, following 8 weeks of resistance training using Zucker diabetic fatty rats (type 2 diabetic animal models).

**[Methods]:**

Twenty-four male lean (Zucker lean control, ZLC) and diabetic (Zucker diabetic fatty, ZDF) rats (age, 8 weeks old) were separated into 3 groups, lean control (ZLC-Con, n=8), diabetic control (ZDF-Con, n=8), and diabetic exercise-trained groups (ZDF-Ex, n=8). The rats in ZDF-Ex were trained to climb a 1-m vertical (85 degrees inclined) ladder with weights. Resistance training was performed with 10 repetitions/day for 12 weeks (3 days/week). The skeletal muscle levels of FGF-21 and irisin were measured using enzyme-linked immunosorbent assays.

**[Results]:**

The levels of FGF-21 in the soleus (SOL) and extensor digitorum longus muscles of ZDF-Ex were higher (p<0.05) compared to levels in ZDF-Con. Additionally, we found a significantly higher irisin level in the SOL muscles of ZDF-Ex compared to that in ZDF-Con. Moreover, we found that the levels of FGF-21 (R=0.532, p=0.02) and irisin (R=0.498, p=0.03) had significant correlations with grip strength.

**[Conclusion]:**

Based on these results, resistance training may be an efficient intervention for increasing FGF-21 and irisin levels in type 2 diabetic (T2DM) skeletal muscles.

## INTRODUCTION

From recent studies, fibroblast growth factor-21 (FGF-21) and irisin have been known to be peroxisome proliferator-activated receptor gamma coactivator 1-alpha (PGC-1 alpha)-related potential therapeutic targets for metabolic diseases. FGF-21, a member of the fibroblast growth factor group, is a well-recognized metabolic and a promising target in managing metabolic diseases. Skeletal muscle-induced FGF-21, which is regulated by a PI3K/Akt1 pathway- dependent mechanism, protects against obesity and insulin resistance, induces the browning of white adipose tissue, and protects against cardiac hypertrophy. With its protecting role against obesity, insulin resistance and even hypertrophy, irisin is known as an exercise-induced myokine that is secreted into the circulation following proteolytic cleavage from its cellular form, fibronectin-type III domain-containing 5 (FNDC5)^1^. It reverses diet-induced obesity and diabetes through stimulating thermogenesis in rodents which increases brown adipocyte-like cell abundance within white fat^2, 3^.

According to a review article by Sanchis-Gomar, PGC-1 alpha-related myokines, including FGF-21 and irisin, could be potential therapeutic targets for metabolic disease and sarcopenia^4^. PGC-1 alpha activation using endocrine activators of brown fat function such as FGF-21 and irisin have been suggested as a beneficial treatment of metabolic disease^5^. Combining these suggestions, this study proposed to investigate the role of the FGF-21-PGC-1 alpha-irisin axis in metabolic disease and sarcopenia. Although the roles of FGF-21 and irisin have been well demonstrated in metabolic disease, there has been no report to investigate the effect of resistance exercise on FGF-21 and irisin levels in diabetic skeletal muscles. Therefore, in the present study, we investigated the change of FGF-21 and irisin levels in various skeletal muscles, and their association with muscle strength, following 8 weeks of resistance training, using Zucker diabetic fatty rats (type 2 diabetic animal models).

## METHODS

### Animals

Male and female Zucker diabetic fatty (ZDF, *fa*/+) rats were purchased from Genetic Models (Indianapolis, ME) and mated with each other. They were housed in a conventional manner, under adequate temperature (23 °C) and humidity (60%) conditions, controlled in a 12-h light/12-h dark cycle, and with free access to food and water. Purina 5008 rodent diets (7.5% fat) were provided as recommended according to Genetic Models Co. (Purina, St. Louis, MO). The genotype of the *fa* gene for these rats was determined using the strategy described in our previous study^6^. Twenty-four male lean (Zucker lean control, ZLC, +/+) and diabetic (Zucker diabetic fatty, ZDF, fa/fa) rats (age, 8 weeks old) were separated into 3 groups, lean control (ZLC-Con, n=8), diabetic control (ZDF-Con, n=8), and diabetic exercise-trained groups (ZDF-Ex, n=8). The training began after one week of adaptation. At 6 weeks (pre-exercise) and 14 weeks (post-exercise) of age, all animals’ body weight (Mettler instrument AG CH-8606, Switzerland), grip strength (Bioseb, France), and fasting plasma glucose levels (Roche Diagnostics LTD., Mannheim, Germany) were measured. The procedures for handling and caring for the animals adhered to guidelines, in compliance with current international laws and policies (NIH Guide for the Care and Use of Laboratory Animals, NIH Publication No. 85-23, 1985, revised 1996), and the protocol was approved by the Institutional Animal Care and Use Committee (IACUC) of Seoul National University (SNU-131007-1). All conducted experiments aimed to minimize the number of animals used and the suffering caused because of the procedures undertaken in the present study.

### Progressive resistance exercise training

The rats in the exercise-trained group (ZDF-Ex) were trained to climb a 1-m vertical (85 degrees inclined) ladder with weights secured to their tail. In the first week, the rats were familiarized with climbing up to the top of the cage with and without weights on their tails. The training sessions, from the second week, started with an intensity at 50% of each rat’s body weight and weights were applied with a conical tube filled with iron pellets securely attached to the tail using a plastic belt and tape. Rats were placed at the bottom of the ladder and obliged to climb to the top. When they reached the top, 2 minutes of rest was allowed before starting the next trial. Subsequent trials were performed from the bottom of the ladder, and a 20-g weight was added to the previous weight in every trial. If a rat could climb 10 times with ever-increasing weights, the training session was considered completed. In cases where a rat failed to complete the climb with increasing weight, the rat was obliged to complete 10 trials with the previous successful weight, with no further attempts made to increase the weight.

### Grip strength test

A grip strength test was conducted using a Grip Strength Meter adapted with a dual grip bar connected to two separate strain gauges to allow separate measurements of force with the two forepaws simultaneously. On each trial, the rat was held around the abdomen and lowered at an angle perpendicular to the bar until it gripped the two bars, one with each forepaw, and with its rear paws standing on the inclined surface of the apparatus. The rat was then pulled gently by the base of the tail, in a rearward direction, away from the bars. Rats instinctively clung to the bar to the point where they could no longer resist the pull. The applied force at the point at which the rat released its grip for each paw was recorded using two separate strain gauges connected to a digital readout. Grip strength was tested in sessions of five trials, separated approximately 1 minute between each trial. The mean of measurements for each rat was used.

### Tissue collection and protein analysis

Two days after the final exercise session, the rats were anesthetized using an IP injection of Zoletil 50 (10 mg/kg, i.p.; Vibac Laboratories, Carros, France). Tissue samples were collected from the gastrocnemius (GAS), soleus (SOL), tibialis anterior (TA), and the extensor digitorum longus (EDL) muscles. Tissue samples were frozen on ice and stored at -80 °C until use. The samples were weighed and then homogenized using an RIPA buffer. Samples were spun at 14,000 r.p.m. for 20 minutes at 4 °C, and the total protein concentration of the supernatant was determined using a Bradford assay. The skeletal muscle levels of FGF-21 (R&D Systems, MN, U.S., Cat. No. MF2100) and irisin (Phoenix Pharmaceuticals, CA, U.S., Cat. No. EK-067-52) were measured using enzyme-linked immunosorbent assays (ELISA), according to the specifications of the manufacturer.

### Statistical analysis

Statistical analysis was performed using the SPSS 18.0 software package. Data were analyzed using a two-sample t-test to examine the levels of FGF-21 and irisin in skeletal muscles. Data are presented as means ± S.E.M. with significance set at *p*<0.05. Associations of FGF-21 and irisin levels in the SOL muscles with grip strength were calculated using Pearson’s correlation coefficient.

## RESULTS

**Figure 1.**
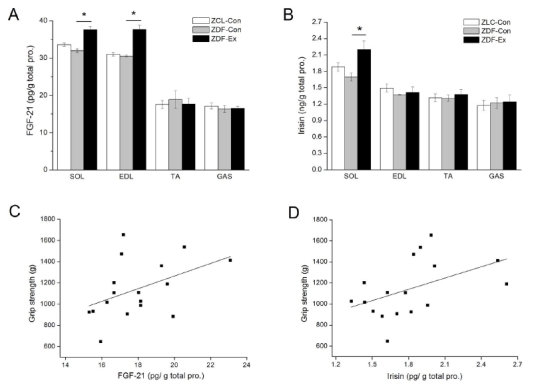
Effect of resistance training on FGF-21 and irisin levels in diabetic skeletal muscles and their associations with grip strength. After 8 weeks of progressive resistance training, the levels of (A) FGF-21 and (B) irisin were measured in SOL, EDL, TA, and GAS muscles. The associations of grip strength with (C) FGF-21 and (D) FGF-21 levels in the SOL muscles were evaluated. A p value *p<0.05 was statistically significant in comparing findings between the ZLC-Con group and the ZDF-Ex group. Pearson’s correlation coefficients and p values are shown in each graph.

The levels of FGF-21 and irisin in the SOL, EDL, TA, and GAS muscles were measured at the end of the 12-week progressive resistance training. In the SOL and EDL muscles, there were no significant differences in FGF-21 levels between ZLC-Con and ZDF-Con. The levels of FGF-21 in the SOL and EDL muscles of ZDF-Ex were higher (*p*<0.05) than the levels in ZDF-Con (Fig. 1A). In addition, we found a significantly higher irisin level in the SOL muscles of ZDF-Ex compared to ZDF-Con (Fig. 1B). However, no significant effect of diabetes and resistance training was detected in either the TA or GAS muscles.

According to our previous report^7^ using the same type of rat, the body weights of the diabetic rats (256.33 ± 5.07 g) were significantly higher than that of the lean control rats (ZDF-Con = 370.04 ± 9.87 g, ZDF-Ex = 341.4 ± 1.21 g). In addition, the grip strength of ZDF-con (3231.84 ± 132.32 g) was lower than that of ZLC-con (2760.14 ± 98.49 g), whereas the grip strength of ZDF-Ex (3191.23 ± 100.31 g) significantly improved compared to the grip strength in the ZDF-Con group^7^. The association between the levels of exercise-induced myokines and grip strength was evaluated in the SOL muscles of the three experimental groups. We found that a level of FGF-21 (R=0.532, p=0.02) and irisin (R=0.498, p=0.03) had significant correlations with grip strength (Fig. C, D).

## DISCUSSION

In the present study, we found an increase of FGF-21 and irisin levels in skeletal muscles (both levels increased in SOL muscles) following 8 weeks of progressive resistance training in T2DM rats. In addition, these increases of FGF-21 and irisin levels in the SOL muscles showed a significant correlation with grip strength after resistance training. This study aimed to investigate the effect of resistance exercise on the level of myokines. Therefore, an improvement of muscle strength could be the result of a suitable application of resistance exercise. In addition, the relationship between grip strength and myokine means myokine levels change due to the individual response of the experiment animal following progressive resistance exercise. To our knowledge, these descriptive results provide the first report on evaluating the effect of resistance training on FGF-21 and irisin levels in skeletal muscles using type 2 diabetic animal models.

Physical activity or exercise training plays a significant role in the prevention and treatment of metabolic diseases. The manner of endocrine secretion occurring with some myokines released during exercise from within skeletal muscle has been considered a potential disease mediator or modulator^8^. There have been previous studies regarding the effect of acute exercise on FGF-21 and irisin circulating levels. In normal mice and healthy humans, increments of serum FGF-21 levels were induced through a single session of treadmill exercise^9^ and through 2 weeks of treadmill training^10^. In addition, the FGF21 circulating level after a 1-hour recovery following a single session of exercise was significantly increased compared to the level prior to exercise, while the level of FGF-21 secreted following high-intensity exercise was higher than that secreted following mild intensity exercise^9^.

Recent research has indicated that circulating irisin is significantly lower in T2DM subjects compared to non-diabetic controls^8,11^. Choi et al. demonstrated significantly decreased circulating irisin levels related to glucose tolerance status in a normal group and in a group with T2DM^12^. Furthermore, high-intensity exercise increased the irisin response compared to low-intensity exercise with similar energy expenditure^13^. In addition, exercise-induced changes of irisin levels did not differ between healthy controls and subjects with metabolic syndrome^14^. However, based on previous reports evaluating irisin level response changes to exercise training, most types of exercise intervention were limited to aerobic exercise. It appears there is a need to investigate the effect of resistance training or to compare the different effects of aerobic and resistance exercise in metabolic diseases.

In this study, we allowed experimental mice free access to food and water. The food intake of the diabetic rats was approximately twice as much as that of the lean control rats. In our results, there was no significant difference between ZDF-con and ZDF-Ex. There was also no significant myokine level difference between the diabetic and lean control rats even though there was a difference of food intake. Additionally, there was no relationship between food intake and myokine level in both diabetic and lean control rat rats (data not shown). However, a limitation of this study was that the causal effect of resistance exercise could not be explained, with only changes in myokine levels following resistance exercise found.

The role of the FGF-21-PGC-1α-irisin axis in age-related conditions including type 2 diabetes was investigated through a possible PGC-α activator such as exercise^4^. Lee et al.,^5^ have suggested that targeting PGC-lα, for example using fat-browning endocrine activators such as FGF-21 and irisin, might benefit in treating metabolic diseases. However, there have been no reports investigating the effect of exercise type (aerobic and resistance exercise) and intensity (vigorous and moderate intensity) on FGF-21 and irisin in metabolic diseases. In this regard, our results may be fundamental in establishing effective exercise standards.
